# Supplementary material for: A simple protein-based surrogate neutralization assay for SARS-CoV-2
Source: JCI Insight. 2020 Oct 2;5(19):e142362. doi: 10.1172/jci.insight.142362 (PMC7566699; doi:10.1172/jci.insight.142362)
Supplement: Supplemental data [file jciinsight-5-142362-s235.pdf]

SUPPLEMENTAL FIGURES

A simple protein-based surrogate neutralization assay for SARS-CoV-2

Kento T. Abe [1,2,•], Zhijie Li [1•], Reuben Samson [1,2], Payman Samavarchi-Tehrani [2], Emelissa J. Valcourt [3], Heidi Wood [3], Patrick Budylowski [4,5], Alan P. Dupuis II [6], Roxie C. Girardin [6], Bhavisha Rathod [2], Jenny H. Wang [2], Miriam Barrios-Rodiles [2], Karen Colwill [2], Allison J McGeer [2, 7, 8, 9], Samira Mubareka [10], Jennifer L. Gommerman [4], Yves Durocher [11], Mario Ostrowski [4,12,13], Kathleen A. McDonough [6,14], Michael A. Drebot [3, 15], Steven J. Drews [16], James M. Rini [1,17*] and Anne-Claude Gingras [1,2*]

[1] *Department of Molecular Genetics, University of Toronto, Toronto, ON, Canada*
[2] *Lunenfeld-Tanenbaum Research Institute at Mount Sinai Hospital, Sinai Health System, Toronto, ON, Canada*
[3] *Zoonotic Diseases and Special Pathogens, National Microbiology Laboratory, Public Health Agency of Canada, Winnipeg, MB, Canada*
[4] *Department of Immunology, University of Toronto, Toronto, ON, Canada*
[5] *Institute of Medical Science, University of Toronto, Toronto, ON, Canada*
[6] *Wadsworth Center, New York State Department of Health, Albany, NY, USA*
[7] *Department of Microbiology, University Health Network and Sinai Health System, Toronto, ON, Canada*
[8] *Dalla Lana School of Public Health, University of Toronto, Toronto, Canada*
[9] *Department of Laboratory Medicine and Pathobiology, University of Toronto, Toronto, ON, Canada*
[10] *Department of Laboratory Medicine and Molecular Diagnostics, Division of Microbiology, Sunnybrook Health Sciences Centre, Toronto, ON, Canada; Biological Sciences, Sunnybrook Research Institute, Toronto, ON, Canada; Division of Infectious Diseases, Sunnybrook Health Sciences Centre; Department of Laboratory Medicine and Pathobiology, University of Toronto.*
[11] *Mammalian Cell Expression, Human Health Therapeutics Research Centre, National Research Council Canada, Montréal, QC, Canada*
[12] *Department of Medicine, University of Toronto, Toronto, ON, Canada*
[13] *Li Ka Shing Knowledge Institute, St. Michael's Hospital, Toronto, ON, Canada*
[14] *Department of Biomedical Sciences, School of Public Health, University at Albany, SUNY, Albany, NY, USA*
[15] *Department of Medical Microbiology and Infectious Disease, University of Manitoba, MA, Canada*
[16] *Canadian Blood Services, Edmonton, AB & Department of Laboratory Medicine and Pathology, University of Alberta, Edmonton, AB, Canada*
[17] *Department of Biochemistry, University of Toronto, Toronto, ON, Canada*

• These authors contributed equally
* Correspondence:
James Rini
MaRS Centre, West Tower, Rm 1614
661 University Ave
Toronto, ON M5G 1M1
416-978-0742
james.rini@utoronto.ca

Anne-Claude Gingras
Mount Sinai Hospital, Lunenfeld-Tanenbaum Research Institute
600 University Ave, Rm 992
Toronto, ON, M5G1X5
(416)450-5417
gingras@lunenfeld.ca

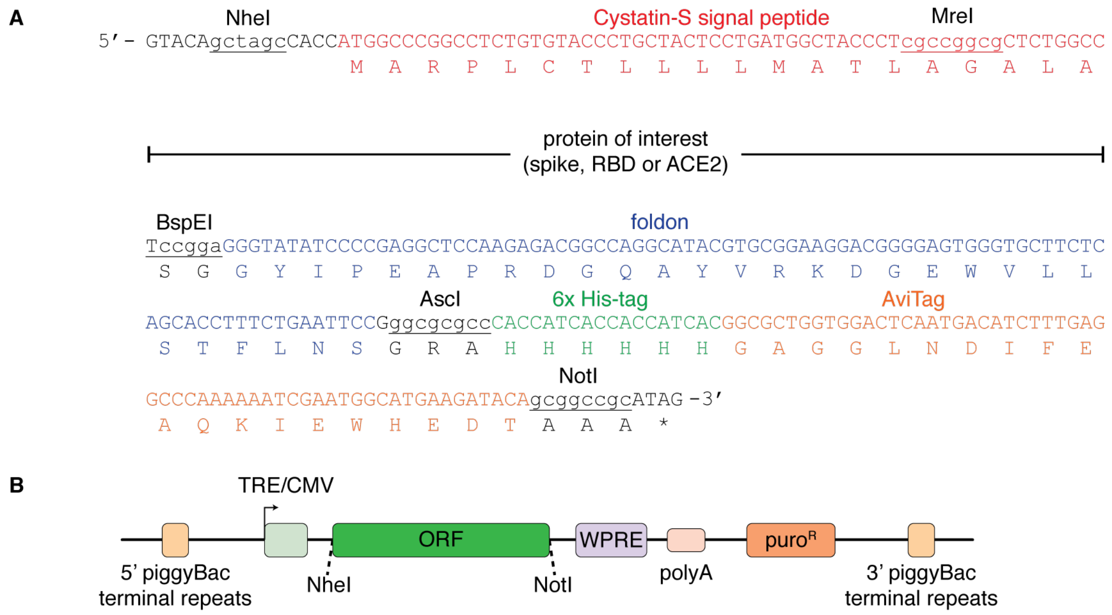

Supplemental Figure 1. Details of the protein expression constructs. (**A**) Multiple cloning site and fusion tags. (**B**) Schematic of the expression vectors used to express recombinant ACE2, spike and RBD.

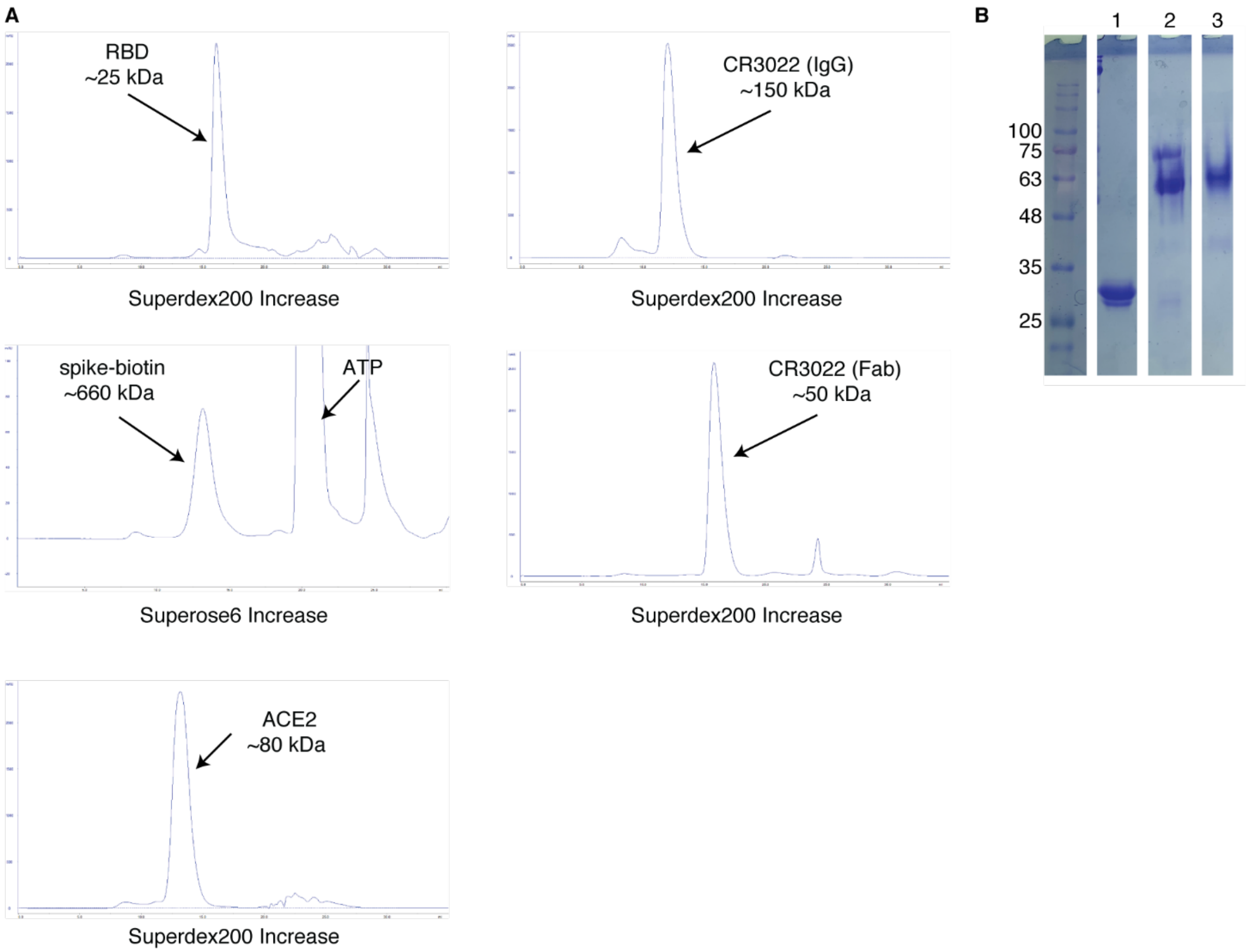

Supplemental Figure 2. Protein purification and biotinylation. (**A**) Size-exclusion chromatography profiles of the RBD, ACE2 and the CR3022 Fab/IgG run on a Superdex 200 Increase column. The spike ectodomain trimer was run on a Superose 6 Increase column. Purification of the spike after the biotinylation reaction is shown to illustrate the separation of the protein from the small molecules. (**B**) Band-shift assay to assess RBD biotinylation: lane 1, SARS-CoV-2 RBD-biotin; lane 2, SARS-CoV-2 RBD-biotin + streptavidin; lane 3, streptavidin.

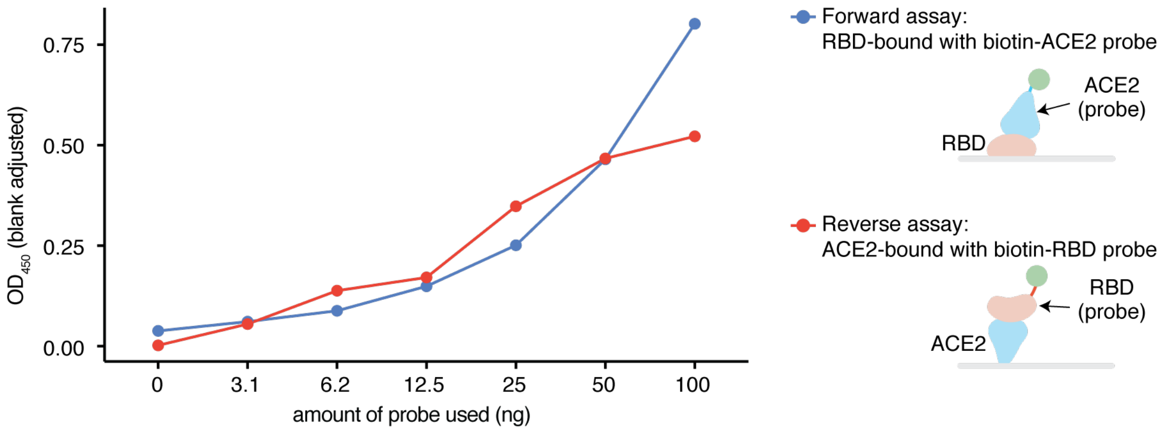

Supplemental Figure 3. Comparison of the signal of the RBD-bound (forward) and ACE-bound (reverse) assays. The forward assay (blue) is where non-labelled RBD is adsorbed onto the ELISA plate and biotinylated ACE2 is added as a probe. The reverse assay (red) is where non-biotinylated ACE2 is adsorbed and probed with biotinylated RBD. After the addition of the probing protein, the presence of the biotinylated protein is evaluated with poly-HRP and TMB. This seven-point curve was generated from one experiment.

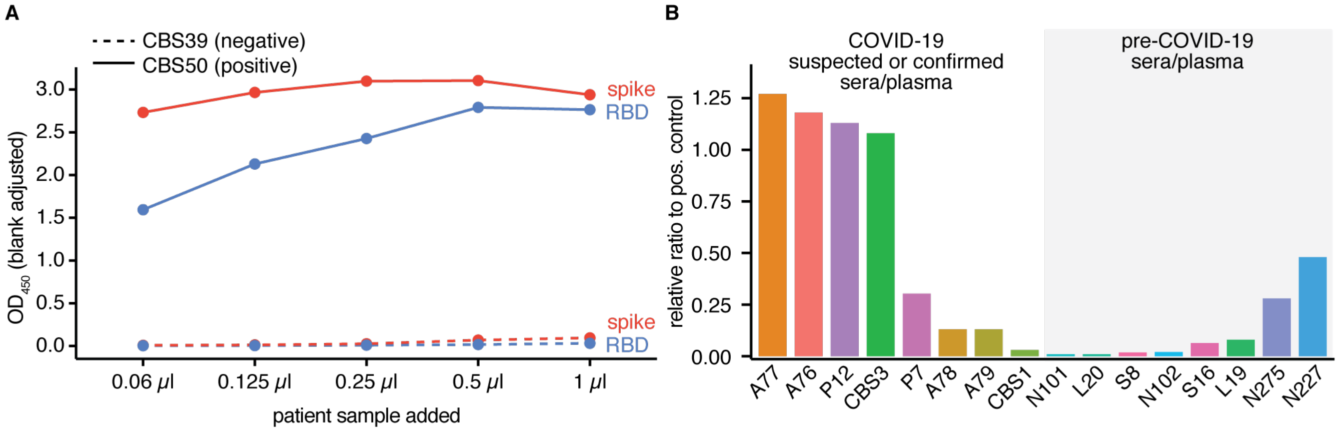

Supplemental Figure 4. Direct binding ELISAs for assay optimization. (**A**) Direct binding ELISA probing for IgG on a dilution series of serum samples CBS39 (negative) and CBS50 (positive). Related to **Figure 1E**. This five-point curve was generated from one experiment. (**B**) Single-point direct binding ELISA assay of 12 pilot samples on the RBD. Data expressed as a ratio to the OD_{450nm} of a convalescent, positive plasma sample used for normalization (P1). The bars represent the mean value from two technical replicates from one experiment. Related to **Figure 1F**.

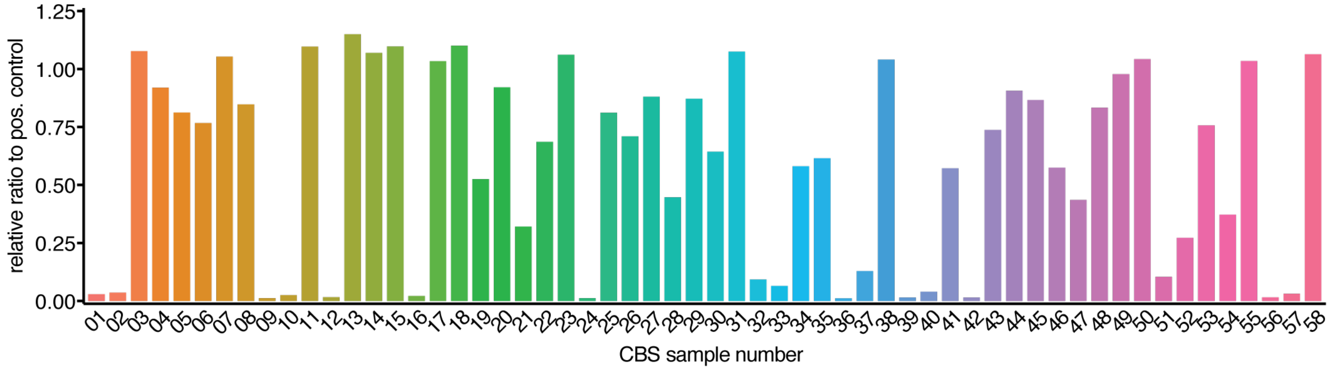

Supplemental Figure 5. Single-point direct binding ELISA assay of all CBS samples on the RBD. Data expressed as a ratio to the OD_{450nm} of a convalescent, positive plasma sample used for normalization (P1). The bars represent the mean from two technical replicates from one experiment (n = 58).

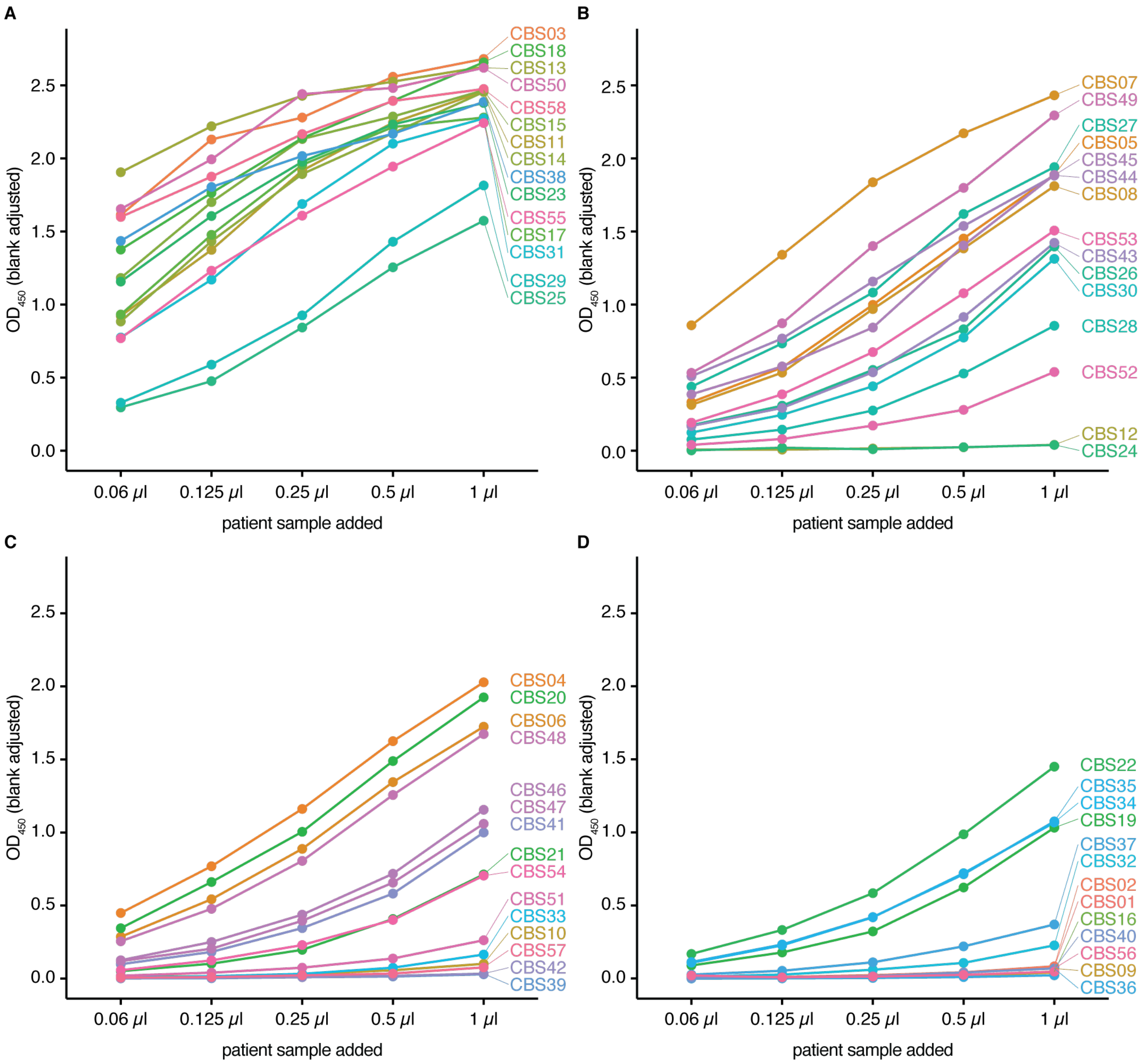

Supplemental Figure 6. The entire set of direct binding ELISAs with titrations on different samples of the Canadian Blood Services cohort. Samples were ranked based on the AUC of the snELISA curve and were plotted in four batches starting with samples most effective at blocking the RBD-ACE2 interaction (**A**), to the lowest (**D**). Related to **Figure 2B** and **Supplemental Figure 8**. The five-point curves were generated from one experiment (n = 58).

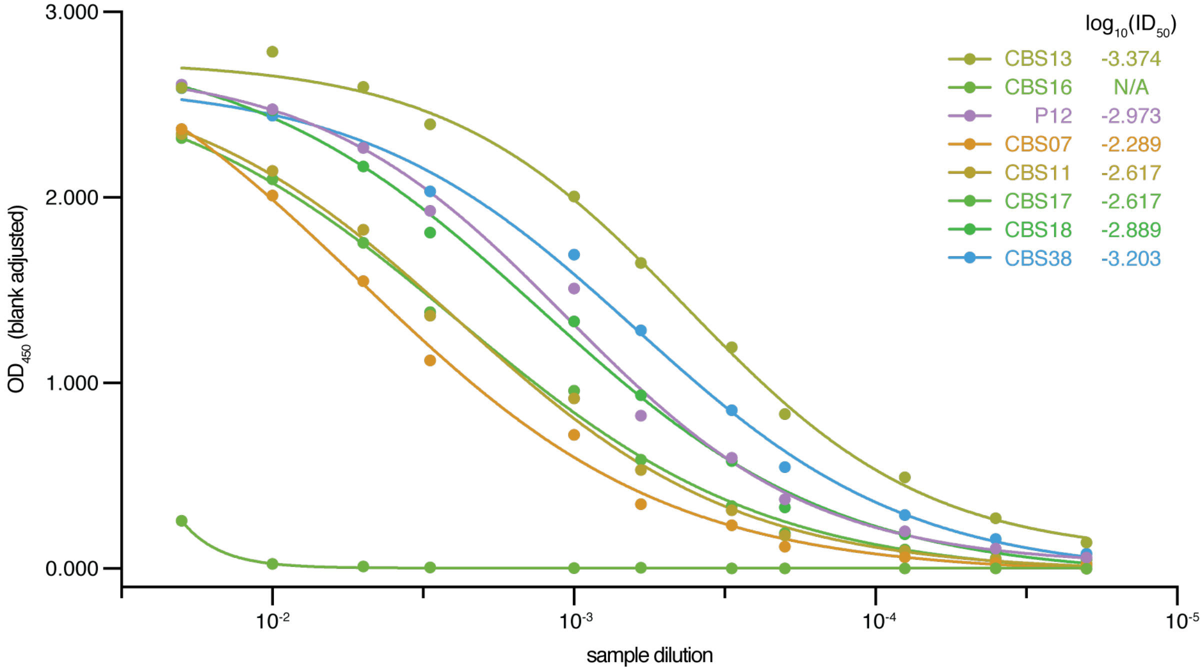

Supplemental Figure 7. An extended direct binding assay dilution series. Six CBS samples exhibiting a high OD_{450nm} at the lower end of the titration curve in the direct binding assay (0.06 µl) were further diluted to generate an extended direct binding titration curve. Related to **Figure 2A** and **Supplemental Figure 6**. The 11-point dilution curves were generated from one experiment.

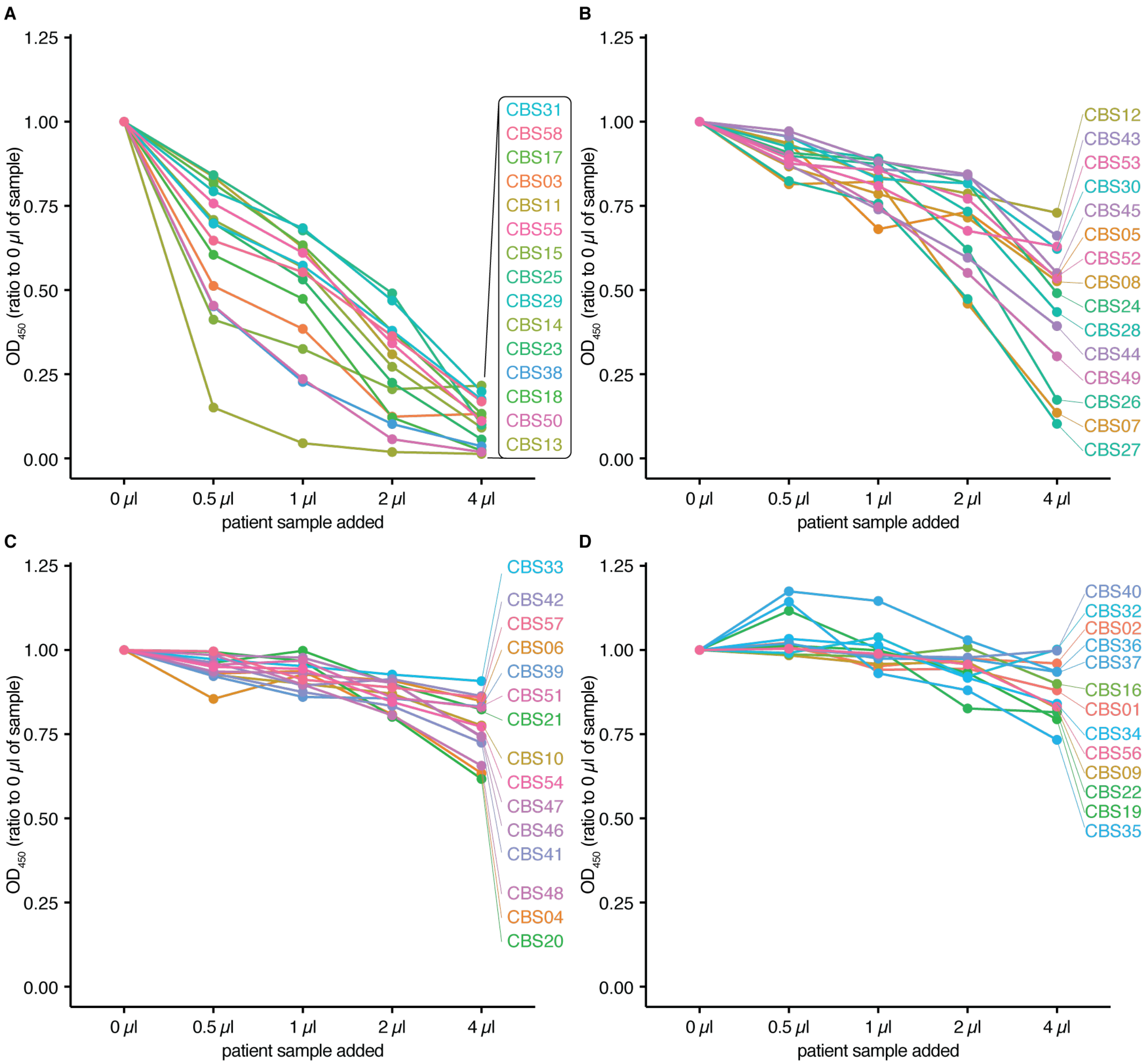

Supplemental Figure 8. The entire set of snELISAs with titrations on different samples of the Canadian Blood Services cohort. Samples were ranked based on the AUC of the snELISA curve and were plotted in four batches starting with samples most effective at blocking the RBD-ACE2 interaction (**A**), to the lowest (**D**). Related to **Figure 2B**. The 5-point dilution curves were generated from one experiment (n = 58).

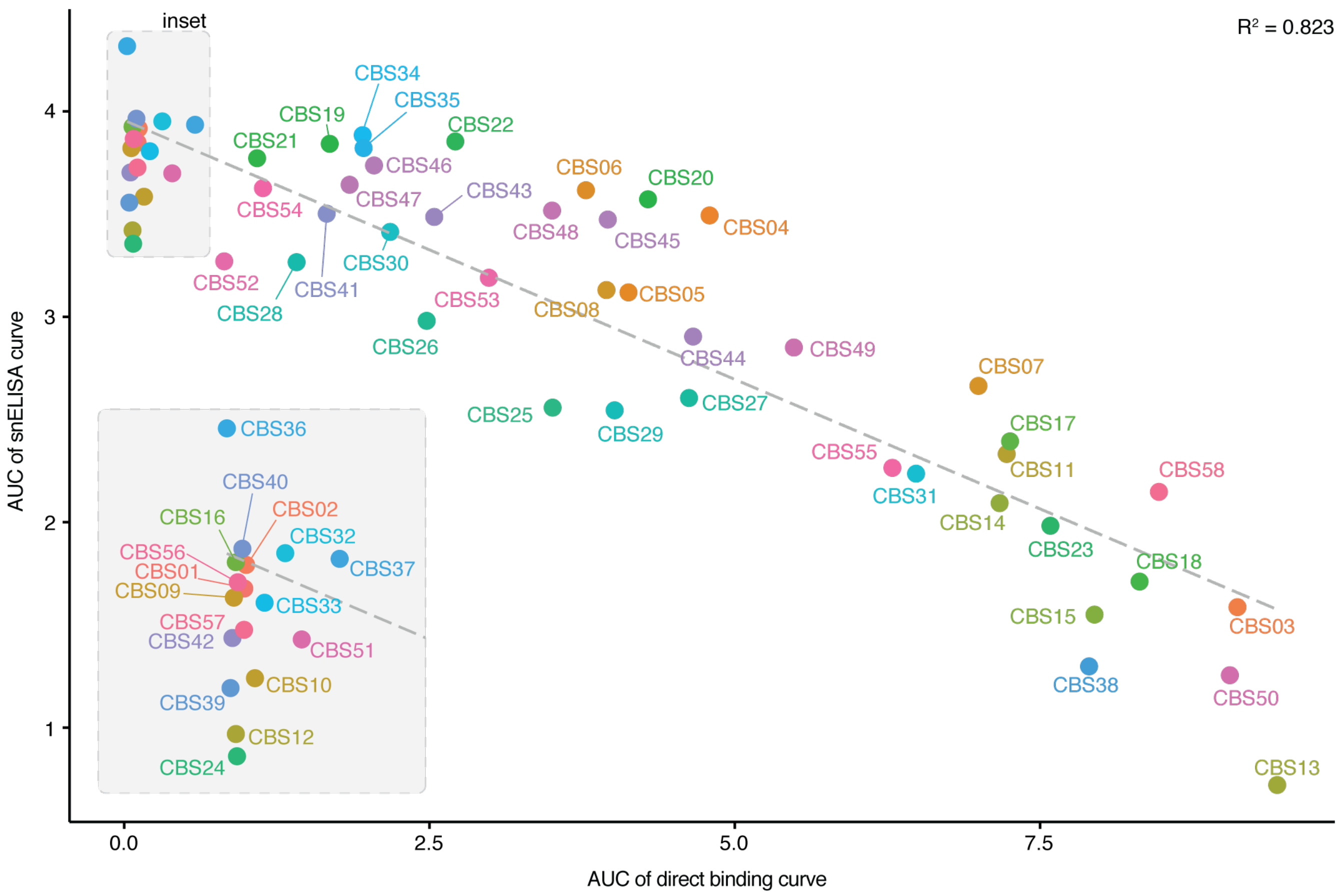

Supplemental Figure 9. Complete correlation plot between the AUCs for the direct and surrogate neutralization ELISAs for all samples profiled with all of the samples labelled. Related to **Figure 2C**.

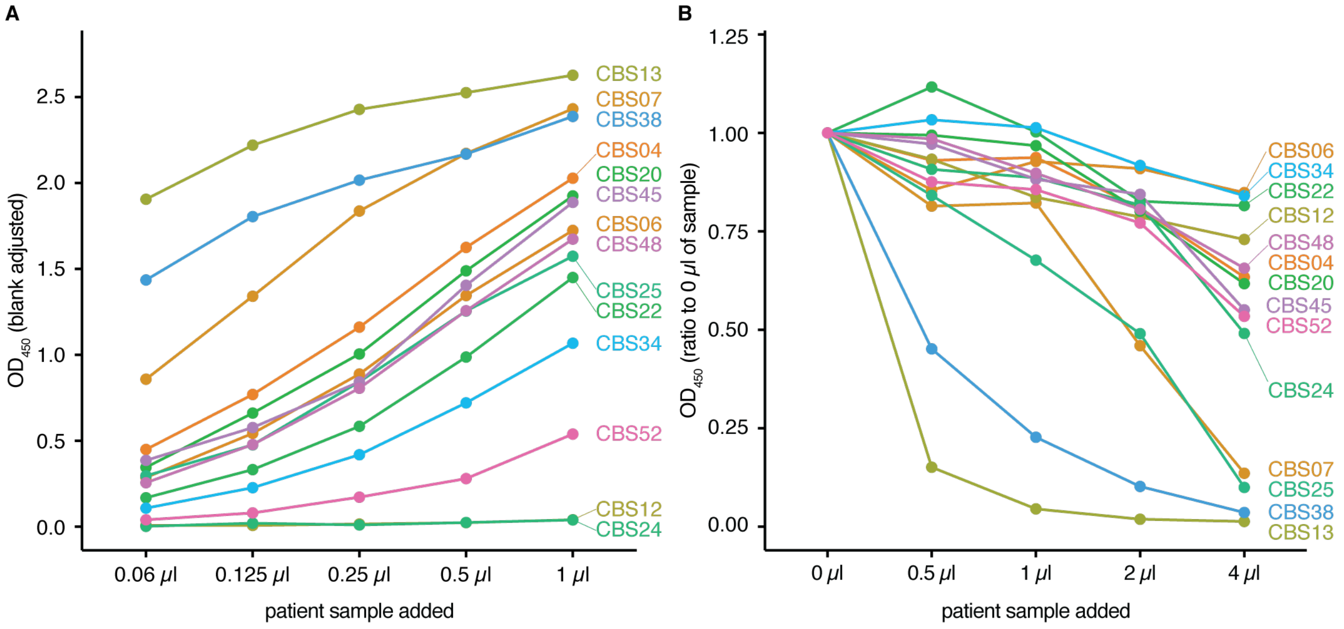

Supplemental Figure 10. ELISA results of CBS samples labelled as outliers (TLS error >0.4). (A) Direct binding assay titration curves and (**B**) snELISA titration curves.

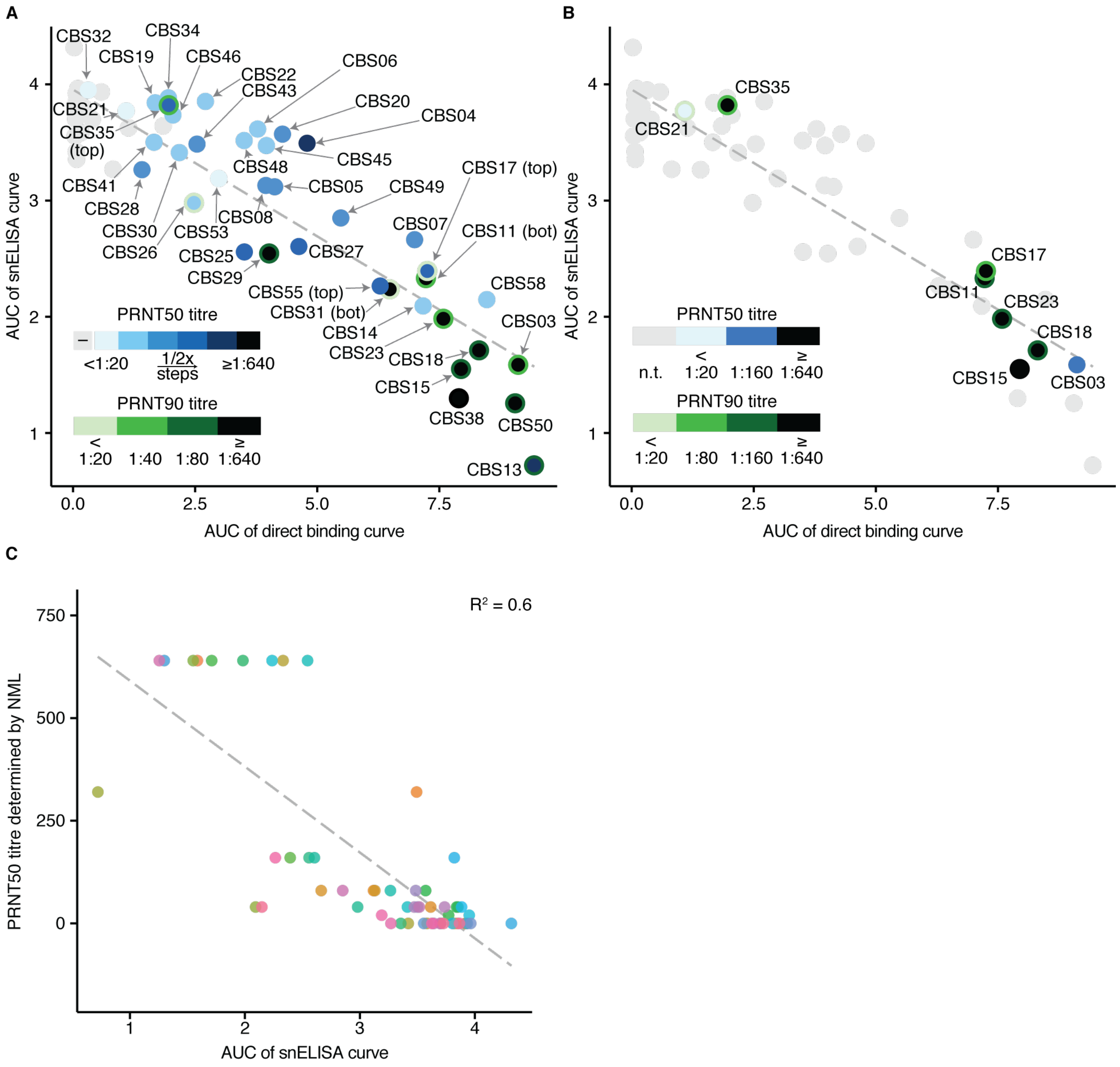

Supplemental Figure 11. PRNT50 and PRNT90 data overlaid on the AUC correlation plot. (**A**) The correlation curve from **Figure 2C** with PRNT50 and PRNT90 data from the NML overlaid on each data point (n = 57). The point fill (blue) represents the PRNT50 titre whereas the outline stroke colour (green) represents the PRNT90 titre. Grey points indicate samples that were negative with both PRNT50 and PRNT90. (**B**) Same as panel (**A**) but with data from Wadsworth (n = 8; samples in grey were not tested). (**C**) Correlation between the results of the PRNT50 titres determined by the NML and the AUC of the snELISA curves calculated for the CBS samples. Note that the titres are binned in 2-fold increments starting with <1:20 and ending with ≥1:640. The grey line represents the linear regression for n = 57 samples. This figure is related to **Figure 2C**.

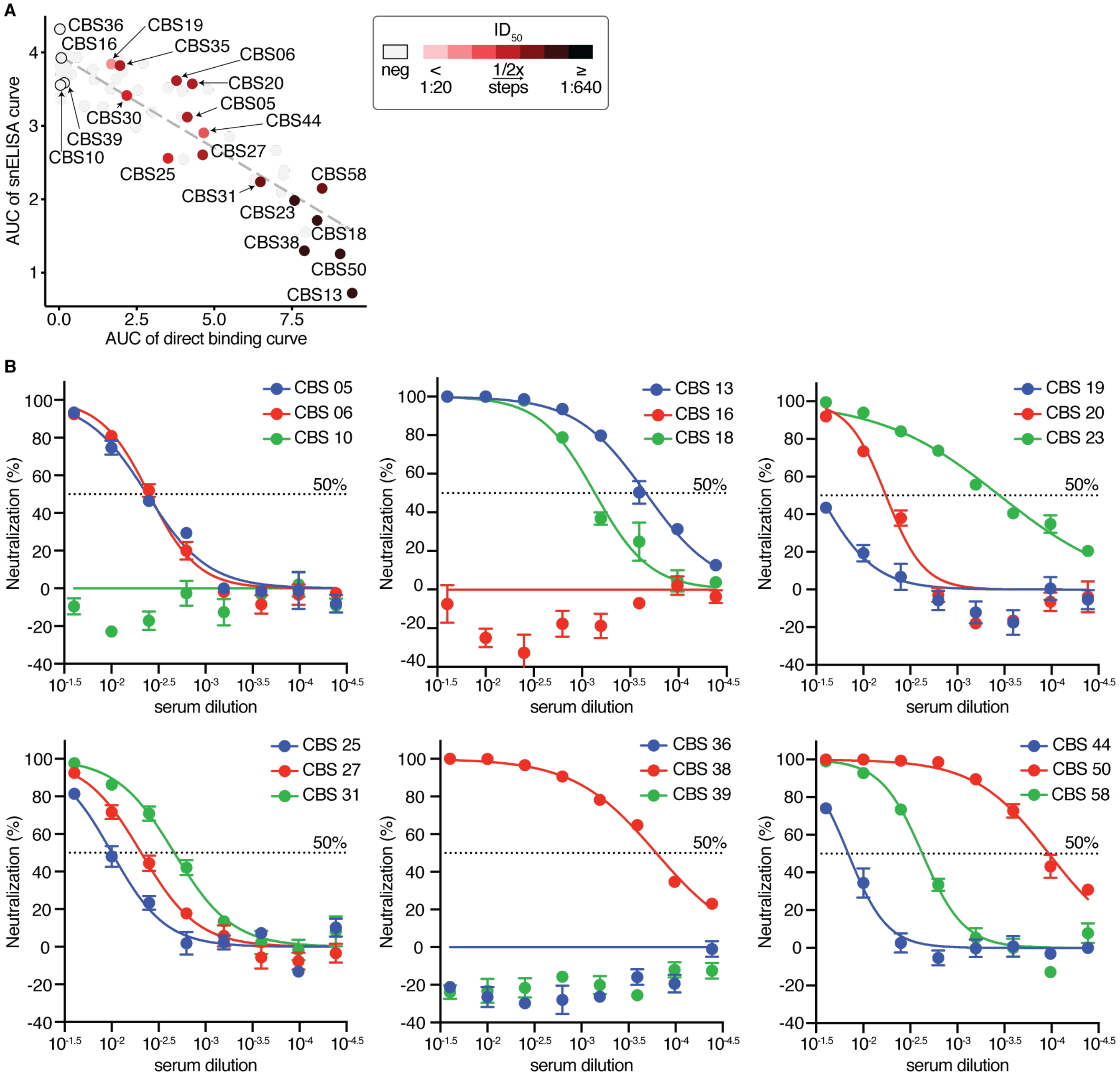

Supplemental Figure 12. Lentiviral spike pseudotyped assay on selected CBS samples. (**A**) The indicated samples were tested by the lentiviral spike pseudotyped assay (n = 21). The ID_{50} of tested samples were calculated and overlaid on the snELISA. Related to **Figure 2C**. (**B**) Neutralization curves used to calculate the ID_{50} values of each sample in **A** (n = 18). Error bars represent the standard error of the mean (SEM) of three replicates.

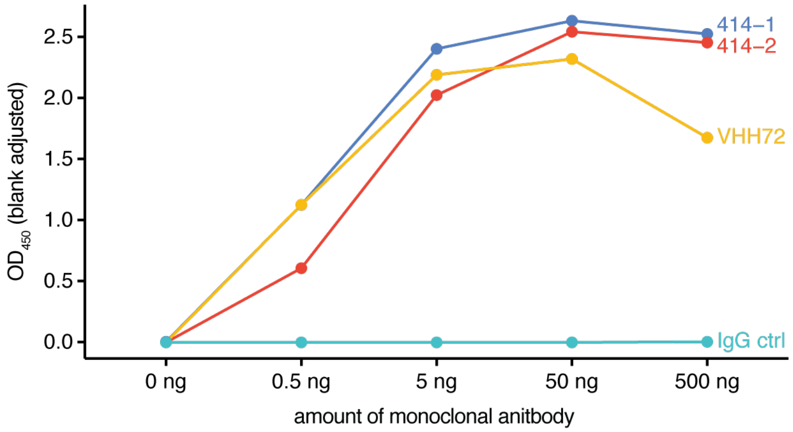

Supplemental Figure 13. Direct binding assay on monoclonal antibodies. Direct binding ELISA probing for IgG on a dilution series of commercial monoclonal antibodies. The 5-point dilution curves were generated from one experiment. Related to **Figure 3C**.

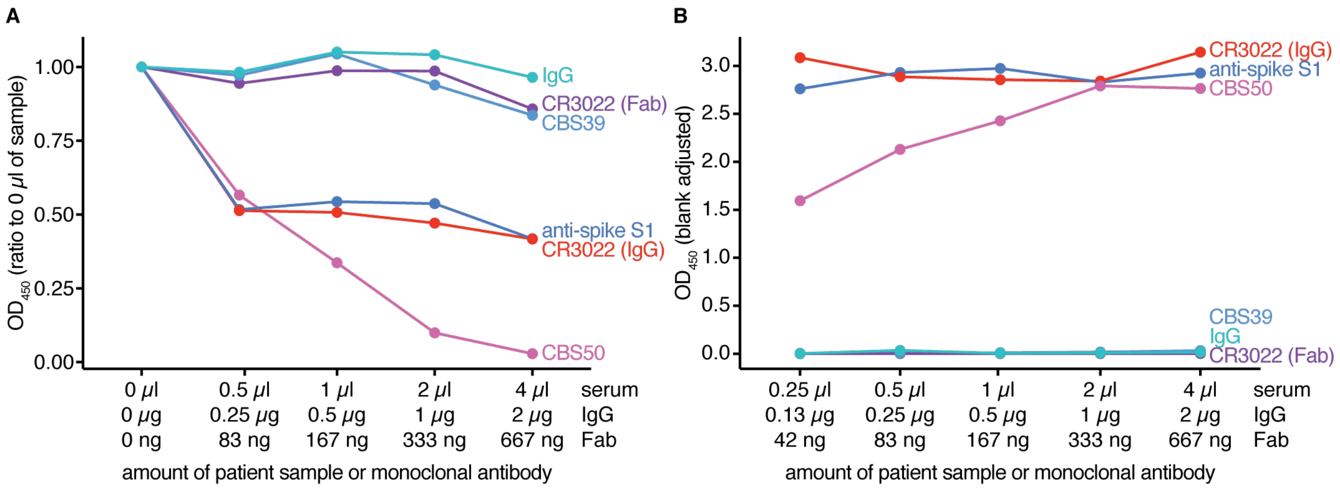

Supplemental Figure 14. Surrogate neutralization and direct binding ELISAs of monoclonal antibodies and affinity reagents. (**A**) snELISA of patient samples and monoclonal antibodies. (**B**) Direct binding assay of a dilution series of the serum samples and antibodies used in **A**. Note that the secondary antibody is an anti-Fc IgG, so the CR3022 (Fab, purple) is not expected to show activity. The 5-point dilution curves were generated from one experiment.

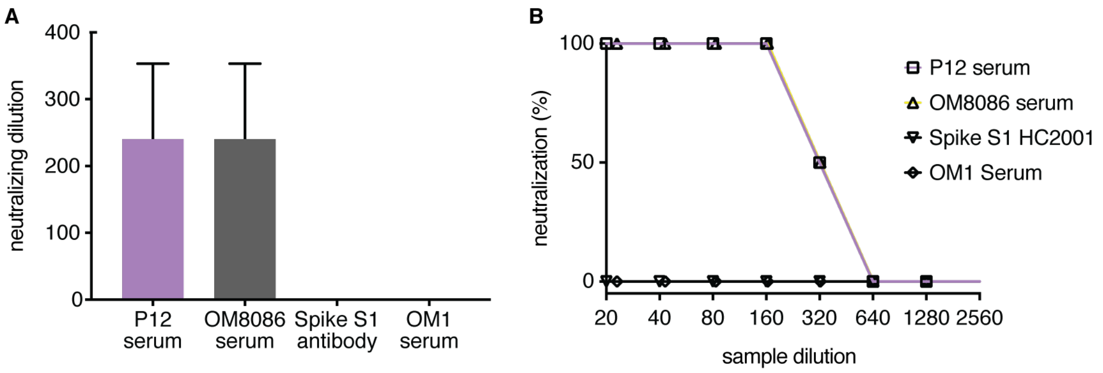

Supplemental Figure 15. Cytopathic effect-reduction neutralization assay on the GenScript anti-spike S1 antibody. Convalescent sera P12 and OM8086 as well as SARS-CoV-2 unexposed serum OM1 and anti-spike monoclonal antibody HC2001 were serially diluted and co-cultured with SARS-CoV-2. The co-cultures were layered onto VeroE6 cells for 1 hr and plaques were monitored. Samples were run in quadruplicates and error bars represent the SEM. (**A**) neutralization dilution; (**B**) percentage neutralization at indicated dilution.

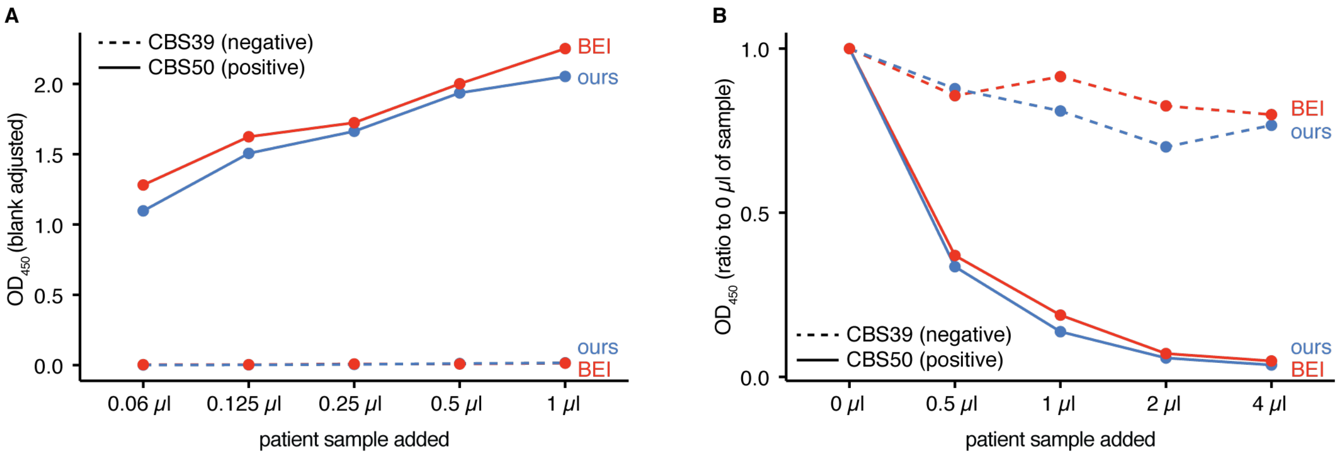

Supplemental Figure 16. Compatibility of the assay with the RBD from a different source (F. Krammer via BEI). Direct binding ELISA probing for IgG on a dilution series of serum samples CBS39 (negative reactivity) and CBS50 (positive reactivity). The 5-point dilution curves were generated from one experiment, and samples were run in technical duplicates.

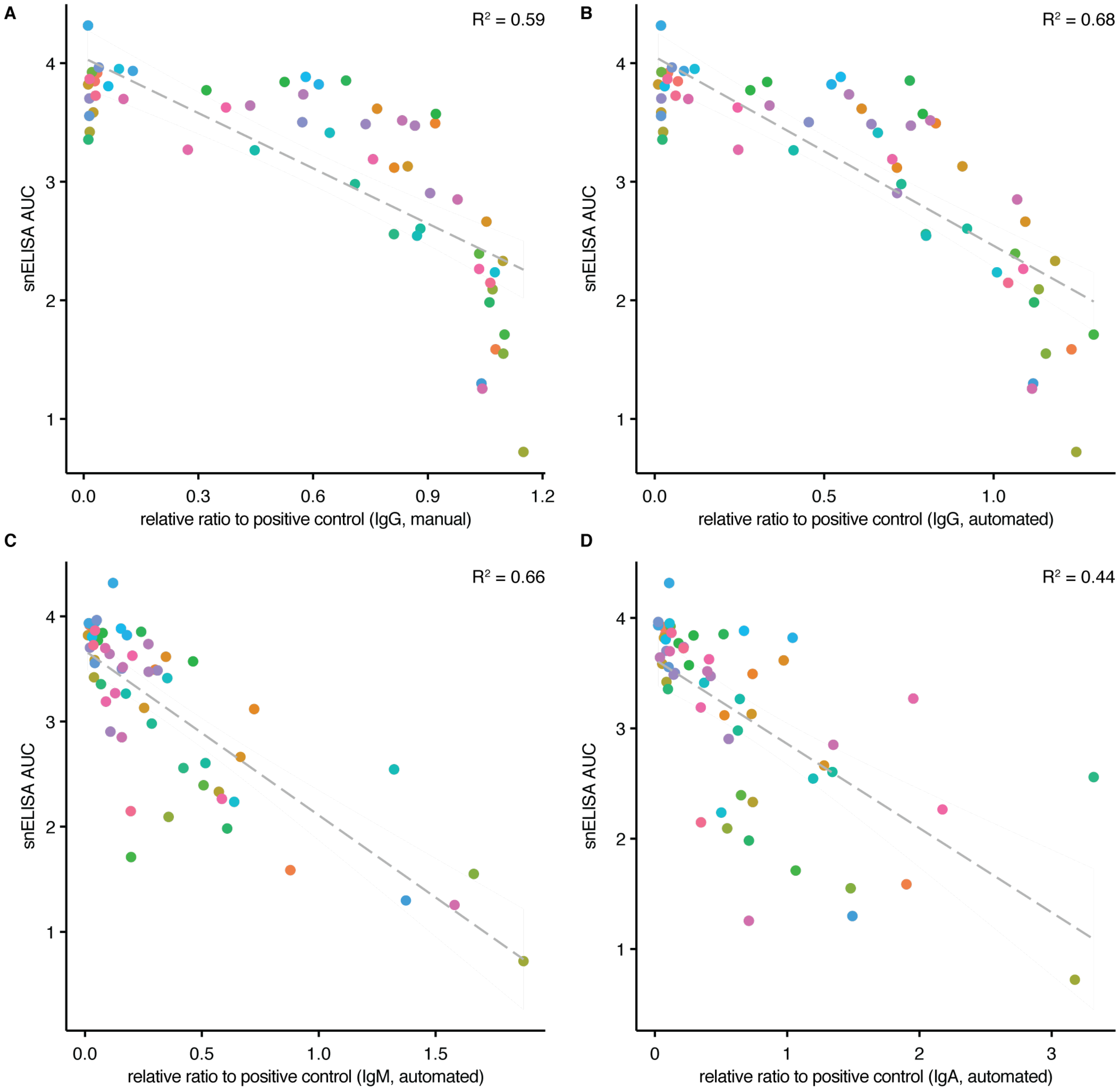

Supplemental Figure 17. Correlation between the results of the snELISA and direct binding ELISAs. The snELISA AUC values were plotted against the single-point direct binding ELISA results probing for (**A**) IgG using data from manual experiments. Panels (**B-D**) show correlations between snELISA AUCs and single-point direct binding results when samples were probed for IgG, IgM and IgA, respectively, using data from ELISAs conducted with an automated platform and a chemiluminescent readout as in (11). Samples (n = 58) were run in technical duplicates, and the grey line represents the linear regression.
